# On the widespread enhancement in fine particulate matter across the Indo-Gangetic Plain towards winter

**DOI:** 10.1038/s41598-020-62710-8

**Published:** 2020-04-03

**Authors:** Narendra Ojha, Amit Sharma, Manish Kumar, Imran Girach, Tabish U. Ansari, Som K. Sharma, Narendra Singh, Andrea Pozzer, Sachin S. Gunthe

**Affiliations:** 1 Space and Atmospheric Sciences division, Physical Research Laboratory, Ahmedabad, India; 2 EWRE Division, Department of Civil Engineering, Indian Institute of Technology Madras, Chennai, India; 3 Atmospheric Chemistry Department, Max Planck Institute for Chemistry, Mainz, Germany; 4 Space Physics Laboratory, Vikram Sarabhai Space Centre, Thiruvananthapuram, India; 5 Lancaster Environment Centre, Lancaster University, Lancaster, UK; 6 Aryabhatta Research Institute of observational sciencES (ARIES), Nainital, India; 7 Present Address: Laboratory for Atmospheric Research, Washington State University, Pullman, USA

**Keywords:** Atmospheric chemistry, Atmospheric dynamics, Atmospheric science, Climate change

## Abstract

Fine particulate matter (PM_2.5_, aerodynamic diameter ≤2.5 µm) impacts the climate, reduces visibility and severely influences human health. The Indo-Gangetic Plain (IGP), home to about one-seventh of the world’s total population and a hotspot of aerosol loading, observes strong enhancements in the PM_2.5_ concentrations towards winter. We performed high-resolution (12 km × 12 km) atmospheric chemical transport modeling (WRF-Chem) for the post-monsoon to winter transition to unravel the underlying dynamics and influences of regional emissions over the region. Model, capturing the observed variations to an extent, reveals that the spatial distribution of PM_2.5_ having patches of enhanced concentrations (≥100 µgm^−3^) during post-monsoon, evolves dramatically into a widespread enhancement across the IGP region during winter. A sensitivity simulation, supported by satellite observations of fires, shows that biomass-burning emissions over the northwest IGP play a crucial role during post-monsoon. Whereas, in contrast, towards winter, a large-scale decline in the air temperature, significantly shallower atmospheric boundary layer, and weaker winds lead to stagnant conditions (ventilation coefficient lower by a factor of ~4) thereby confining the anthropogenic influences closer to the surface. Such changes in the controlling processes from post-monsoon to winter transition profoundly affect the composition of the fine aerosols over the IGP region. The study highlights the need to critically consider the distinct meteorological processes of west-to-east IGP and changes in dominant sources from post-monsoon to winter in the formulation of future pollution mitigation policies.

## Introduction

The Indo-Gangetic Plain (IGP) region is considered to be a global hotspot of elevated aerosol loading, and is among few regions of the world, which have been experiencing enhancements^1–4^. Atmospheric processes occurring in this region have been shown to affect atmospheric composition, chemistry and climate over regional as well as global scales^5–8^. Fine particulate matter (PM_2.5_) in this region is estimated to cause severe health implications including premature mortalities^9–12^. Numerous efforts based on *in-situ* and satellite-based observations have revealed strong enhancements in the PM_2.5_ over this region towards winter, typically every year^13–16^. PM_2.5_ concentrations over the IGP region are observed to exceed the standards of World Health Organization (WHO) as well as the National Ambient Air Quality Standards (NAAQS) of India, attributed to the combined effects from a variety of anthropogenic and biomass-burning emissions and meteorological conditions^17–24^. The accumulation of aerosols in this region is further supported by the topography of Himalaya that extends from northwest to southeast and forms a corridor along the IGP.

The changes in meteorology and atmospheric dynamics can offset the benefits availed from reduction in anthropogenic emissions. Such meteorological influences, besides the elevated emission source strengths and upwind biomass-burning, highly complicate the detailed understanding of aerosol behavior over the IGP^18,22–24^. The implementation of an odd-even scheme for vehicular traffic aiming to reduce its emissions in Delhi, India’s capital territory, did not lead to a significant improvement in the air quality^25,26^, which highlighs the crucial need of studies on the spatio-temporal distribution of PM_2.5_ over the complex environment of IGP region. Surface-based measurements did reveal the impacts of meteorology on aerosol properties over this region, however, such point observations are representative of a limited area and hence need to be corroborated with model results^27^ for portraying the regional picture. In this direction, a global model has been applied to explore the influences of meteorological conditions on the spatio-temporal distribution of PM_2.5_ over this region, recently^23^.

**Figure 1:**
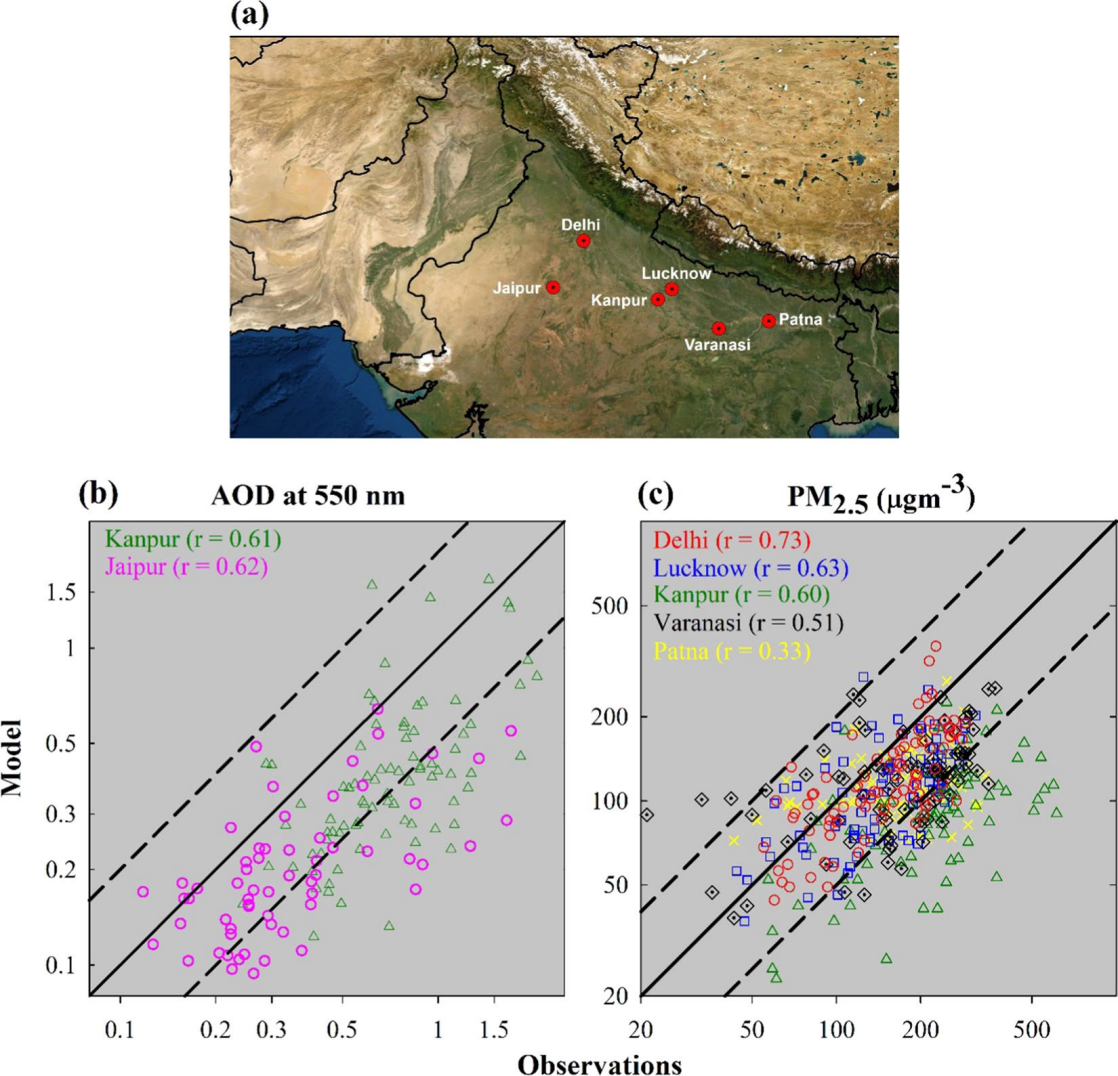
(**a**) The study region (also the domain of the regional model WRF-Chem), and the locations of observation sites used in the study. Correlation between the model and observations for the day-to-day variations in (**b**) AOD at 550 nm, and (**c)** PM_2.5_ during October-December 2016. Solid black line shows a 1:1 relation and dashed lines show an agreement within a factor of 2.

Regional modeling studies, exploiting the potential of high-resolution input emissions together with a well-resolved meteorology^28–32^, are essential to unravel the effects of dynamics and emissions (biomass-burning versus anthropogenic) on the widespread PM_2.5_ build-up across the IGP from post-monsoon to winter transition. In this direction, here we performed high-resolution (12 km × 12 km) simulations using the Weather Research and Forecasting model coupled with Chemistry (WRF-Chem)^33^, configured on the basis of the previous evaluations of meteorology, wind patterns, and chemical fields over the South Asian region^30,32,34,35^ (Table S1). Figure 1a shows the study region (also the domain of the WRF-Chem model), and the location of ground-based observations. Model simulations are performed for the period of October–December 2016, on the basis of previously reported extremely high fine particulate loading during the post-monsoon and winter over the IGP region^36–38^. Besides the reference simulation, called as “WRF-Chem”, two additional simulations have been performed by alternatively switching off the anthropogenic (anthro_off) and biomass burning (fire_off) emissions in the model.

## Results

### Aerosol distribution: Model versus observations

Aerosol optical depth (AOD) at 550 nm is simulated to be higher over the IGP region (0.5–1) in the results of the standard simulation, which is in good agreement with the satellite-based observations from moderate resolution imaging spectroradiometer (MODIS) and multiangle imaging spectroradiometer (MISR) (Fig. S1). The domain-wide mean AOD values simulated by the model (e.g. 0.27 ± 0.14 in October) are found to be comparable with that in the MODIS and MISR observations (0.29 ± 0.21 and 0.24 ± 0.14, respectively for the same period). Some differences are, however, seen not only between model and satellite, but also within different satellite-based instruments, as reported for retrievals of other trace species over this region^29^. Nevertheless, the spatial variability between model and satellite observations is found to be correlated with *r* values of 0.67–0.74 (with MODIS) and 0.71–0.85 (with MISR) during different months over the period of study. Further, model captures the variations in the AOD (r = ~0.6) at Kanpur and Jaipur stations as obtained from the Aerosol Robotic Network (AERONET) observations in this region, to a reasonable extent (Fig. 1b) with underestimated magnitudes. AOD uncertainties in the model are also found to be associated with the dust transport from the domain boundaries (based on additional sensitivity simulations with varying dust in the boundary conditions, not shown here). Nevertheless, the dust transport from domain boundaries is seen to cause lesser impacts on the surface level PM_2.5_ in the simulations (also see the section-Methods). Additionally, the model simulated day-to-day variations in the PM_2.5_ concentrations correlate with the ground-based observations at Delhi, Lucknow, Kanpur, Varanasi, and Patna stations in the IGP region with *r* values in the range of 0.33–0.73 (Figs. 1c and S2). However, model tends to underestimate the PM_2.5_ levels with Normalized Mean Biases (NMB) in the range of 21–35%, except at an industrial site Kanpur in the central IGP where analogous to the AOD, PM_2.5_ levels were underestimated more strongly (NMB = 59%). Model is seen to reasonably capture the temporal variations and magnitudes, and stronger bias at one of the stations is also within the range of model bias (40–60%) reported in an earlier evaluation of this model over this region during winter^28^. The underestimations of aerosol loadings by model are suggested to be resulting from the uncertainties associated with the input emissions^28,39^ and fine-scale dynamics^30,40^, besides some small-scale fires and local sources such as trash and wood burning unaccounted or underestimated in the inputs. Furthermore, models can have a dilution effect over grid boxes as compared to the observations sampling directly the urban and rural air masses at a location/point, which we have tried to minimize by performing simulations at higher spatial resolution (12 km × 12 km). Overall, the WRF-Chem model shows the ability to simulate the distribution of aerosols over northern Indian region, besides some differences in the magnitudes attributed to the aforementioned factors.

**Figure 2:**
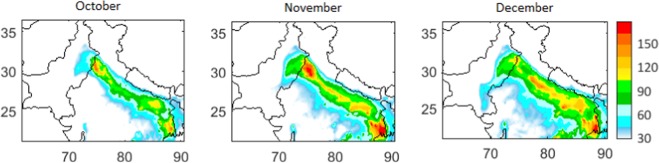
Mean distribution of PM_2.5_ (µgm^−3^) over the northern Indian region during October, November, and December 2016 as simulated by the WRF-Chem model.

The model, capturing the observed variations, reveals strong heterogeneity in the PM_2.5_ distribution over the northern Indian subcontinent (Fig. 2) with elevated levels (100–200 µgm^−3^) over the IGP region. During October, high PM_2.5_ levels (≥100 µgm^−3^) were most pronounced over the western IGP including Delhi, Haryana, and Punjab. Additionally, in the same period, similarly high PM_2.5_ concentrations are simulated over the eastern parts of the IGP. A uniform extension of high PM_2.5_ concentrations (100–200 µgm^−3^) is evident across the entire IGP region during November–December. The model also simulates a distinct pool of strongly enhanced PM_2.5_ (100–150 µgm^−3^) over the eastern IGP (Eastern Uttar Pradesh, and Bihar provinces of India) throughout the study period, despite lower emissions than the northwest and southeast IGP region. Such feature was earlier seen in the satellite-based observations and called as the ‘aerosol pool’ or ‘Bihar pollution pool’^2,36^. As the model is able to simulate the spatio-temporal characteristics of the PM_2.5_ distribution over this region, we analyze model results in greater detail to investigate the influences of various underlying processes and factors such as the meteorology, dynamics, and emissions.

### Influences of meteorology and dynamics

**Figure 3:**
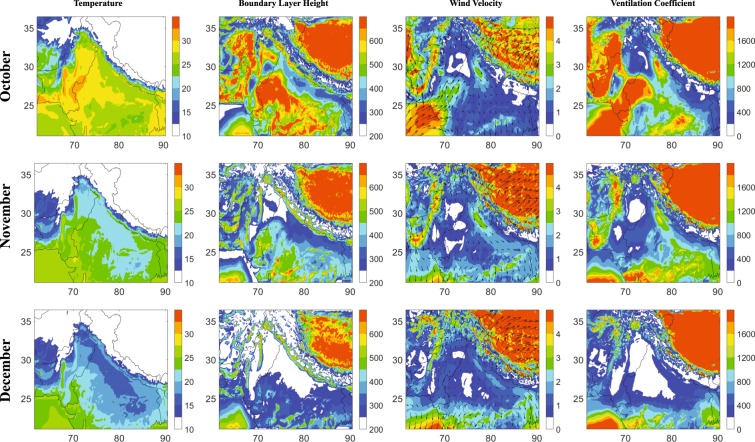
Near surface temperature (°C), boundary layer height (m), mean winds (ms^−1^), and ventilation coefficient (m^2^s^−1^) over the northern Indian subcontinent during October, November and December 2016, based on the WRF-Chem model.

Model simulated meteorological fields reveal strong spatio-temporal variability over the northern Indian subcontinent during October-December (Fig. 3). Higher temperature (28–33 °C) were simulated over the Punjab and Sindh regions of northwest IGP during October, whereas, temperature over other parts of the IGP were generally lower (25–28 °C), except over some parts of the eastern IGP (West Bengal, India and Bangladesh). Temperature shows a decline in November (20–25 °C) and December (15–20 °C) with nearly similar distribution over the western and central IGP, while the eastern IGP experiences slightly higher temperatures than other regions during November-December. These changes in the temperature are correlated with the changes in the boundary layer dynamics across the region, with higher boundary layer height (BLH) during October (>300 m), deeper BLH being confined to the western IGP over Pakistan (>450 m), while, shallower over the eastern IGP (<450 m). Changes in the meteorological and dynamical conditions lead to the suppression of boundary layer to as low as 200–250 m over the western IGP and 250–350 m over central and eastern IGP in November. The western IGP depicts BLH in the range of 200–250 m during December, while the BLH over the central IGP is 200–300 m and eastern IGP is 250–350 m. Model simulates weak north-westerlies (~1 ms^−1^) during October with corresponding ventilation coefficients^41^ (VC) of about 600–1000 m^2^s^−1^ over central IGP and a stronger atmospheric dispersion (>1000 m^2^s^−1^) surrounding India’s capital region of Delhi. A slight increase in the wind speed (1.5–2.5 ms^−1^) is revealed from October to November, besides an enhanced wind zone in the northern India (2.5–3.5 ms^−1^). With decrease in the wind speed from November to December, a significant shallowing of the boundary layer drastically reduces the ventilation to the minimum (VC: < 400 m^2^s^−1^) over most of the IGP.

WRF-Chem simulated wind vectors describe the atmospheric dynamic that facilitates the accumulation of fine aerosols over the IGP. Divergence over the northwestern IGP (region of strong biomass-burning emissions) and concurrently weaker winds over the central and eastern IGP allow the accumulation of fine aerosols from northwest, further downwind (central and eastern IGP), favoring its mixing with local anthropogenic influences. However, during December, reduction in BLH along with wind speeds brings down the ventilation coefficient over the IGP enhancing the PM_2.5_ dramatically. An analysis of vertical distribution of PM_2.5_ and wind speed (Fig. S3) shows that the vertical extent of higher aerosol concentrations is reduced during December with a concurrent reduction in the wind speed from surface to ~875 hPa. A weakening of winds towards December is seen aloft (Fig. S4) resulting in less efficient dispersion of aerosols accumulated in the planetary boundary layer over the IGP.

Meteorology and regional-scale dynamics is suggested to play the crucial role in the widespread PM_2.5_ build up across the IGP in December. Such changes in the meteorological conditions reducing drastically the ventilation across the IGP are typically seen every year (e.g. Fig. S5). Aerosol-radiation interactions are suggested to reduce the boundary layer evolution over this region by reducing the incoming solar radiation reaching surface, and such a feedback can further increase the aerosol concentrations near the surface^42^. The relative importance of the biomass-burning emissions compared to the anthropogenic emissions is analyzed next, based on two sensitivity simulations.

### Influences of emissions: Biomass-burning versus anthropogenic

Biomass-burning emissions, in particular from the open burning of the agricultural residues, significantly enhance the aerosol loading over the IGP region during the study period, in addition to strong regional anthropogenic emissions^18,22,24^. Satellite-based observations (Fig. S6) clearly show strong biomass-burning activities over the northwest IGP during October, whereas, scattered fire activities with lower number of the total events are observed during December. Such distributions of the fire events are not specific for the year 2016 and that fire activities over this region are found to be similar in other years as well (fire activities for years 2015 and 2017 are also shown in the Fig. S6).

**Figure 4:**
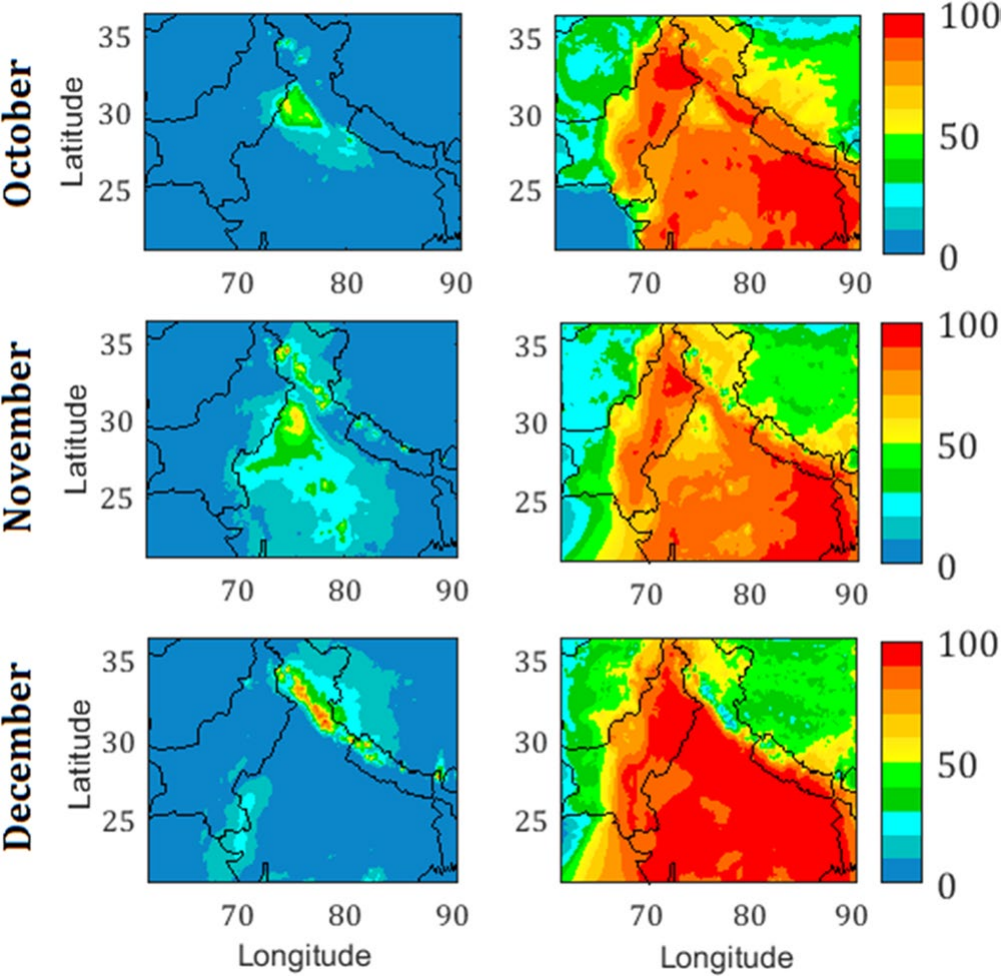
Reduction in PM_2.5_ (%) concentrations due to switching off the biomass-burning (left panel) and anthropogenic emissions (right panel) as compared to the reference simulation.

Figure 4 shows the percentage reductions in the PM_2.5_ concentrations due to switching off the biomass-burning and anthropogenic emissions as compared to the reference simulation. A significant reduction in the PM_2.5_ (by up to ~50–60%) is simulated over the northwest IGP region during October in absence of the biomass-burning emissions, e.g., over Haryana (40–50%), Punjab (50–60%), followed by Delhi National Capital Region (NCR) and the adjoining areas. Model reveals that biomass-burning emissions influenced the PM_2.5_ over larger areas of Punjab and the entire north-central Indian region during November. Analysis of back air trajectories demonstrates that the fire influences are efficiently transported to Delhi during October as well as November (Fig. S7). Whereas, airmasses may not pick maximum effects from these fires to the central and eastern IGP regions in October; but with the change in synoptic-scale winds in November fire effects are transported across the IGP; and aerosols are also dispersed to the central India and surrounding wider region (Figs. S7 and 4). Interestingly during December, when enhanced PM_2.5_ is most widespread, only minimal reduction (less than 10%) is simulated over IGP, without biomass-burning emissions. Nevertheless, fires are found to play major influences over the hills located in the north of IGP in India and parts of Nepal during December which is found to be in agreement with satellite-retrieved fire observations (Fig. S6). The effects of observed forest fires in the sub-Himalaya during December are most pronounced within that region itself with lesser effects being transported to the IGP (Figs. S7 and 4). By switching off the anthropogenic emissions, very large reductions (80–100%) in PM_2.5_ are simulated over nearly the entire IGP region, except the northwest IGP (40–70%) during the post-monsoon, complementing the strong influence of the biomass-burning emissions (noticed in the left panel of Fig. 4). Anthropogenic emissions are found to influence the entire IGP region by 90–100% during December.

**Figure 5:**
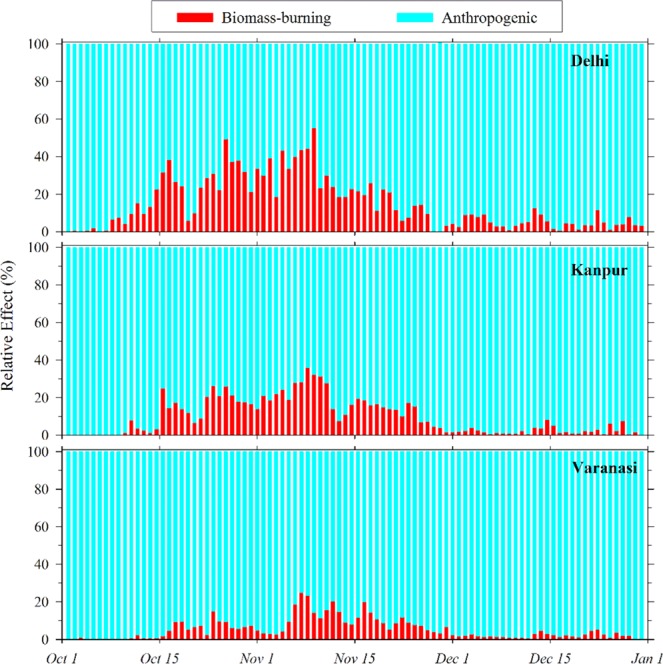
Relative effects of regional biomass burning and anthropogenic emissions on the modeled PM_2.5_ at selected stations in the IGP region.

Individual influences caused by switching off the biomass-burning and anthropogenic emissions on the PM_2.5_ levels are further combined to compute their relative effects (normalizing with the sum of their individual effects). Figure 5 shows the day-to-day variations in the relative effects from the regional biomass-burning and anthropogenic emissions on PM_2.5_ variations at selected locations (Delhi, Kanpur, and Varanasi) in the IGP from west to east. Model results exhibit strong effects of fire emissions on PM_2.5_ at Delhi (daily values up to 55.4%) during the mid-October to mid-November, attributed to the upwind biomass-burning (Figs. S6 and S7). Such influences from biomass burning are also considerable during this period at other locations in the IGP, nevertheless, effects are found to be lower (up to 36.3%) than that over Delhi. During this period of peak fire influences, the mean relative effects from the biomass burning are estimated to be 30.2%, 19.6%, and 9.4% at Delhi, Kanpur, and Varanasi respectively. However, the impact of biomass-burning decreases gradually from mid-November onwards at all the stations attaining minimal values (~5% and less) during December. Concurrently, the relative effect of anthropogenic emissions at all the stations increased to 95% or more during December.

Further, the influences of anthropogenic and biomass burning emissions on the chemical constituents of PM_2.5_, e.g., nitrate (NO_3_^−^), sulphate (SO_4_^−2^), ammonium (NH_4_^+^), organic matter (OM) and elemental carbon (EC) are examined. The differences in the concentrations of these species in absence of fire (Fig. S8) and anthropogenic emissions (Fig. S9) are computed as compared to those in the reference simulation. Reductions by up to ~20 µg/kg dry air (written as µg/kg hereafter) in NO_3_^−^ are simulated in absence of the fire emissions over northwest IGP during October, which spread to larger region during November. Similarly, considerable reductions in the OM (by up to ~50 µg/kg) and EC (by ~10 µg/kg) are also simulated in absence of fires. These results clearly depict that composition of PM_2.5_ aerosols over the IGP region is significantly affected by the biomass burning emissions. The contributions of biomass burning emissions to NO_3_^−^, NH_4_^+^, OM, and EC are seen to drop dramatically during December. In contrast, the secondary inorganic species (NO_3_^−^, SO_4_^−2^, and NH_4_^+^) typically show major influences from the anthropogenic emissions (Fig. S9). Observations showing strong role of secondary sources at megacity Delhi during colder conditions^43^ also highlight the importance of meteorology and regional-scale air chemistry, besides emissions in governing the air quality over the IGP region.

## Discussion

Our study shows that both the meteorological processes (winds, temperature, boundary layer mixing) as well as relative influences of emissions (biomass-burning versus anthropogenic) change drastically during the post-monsoon to winter transition, leading to the widespread enhancement in PM_2.5_ levels across the IGP region. In addition to previous studies, e.g.^15,28,38^, here the inter-dependence of PM_2.5_ distribution, meteorology, and atmospheric chemistry is explored further in impacting the regional air quality based on the high-resolution modeling of PM_2.5_ and constituents. The findings have important implications for the air quality in this densely populated region, which gets severely degraded during post-monsoon and winter seasons. Based on temporal changes in the dominance of biomass-burning and anthropogenic emissions, the study addresses their relative roles in building-up short-term dense haze over the IGP, elucidated in earlier ground-based studies^18,20,22^. Our current findings are also in line with the wintertime abundance of volatile organic compounds over IGP^29^ – which can act as aerosol precursors. In addition, the South Asian outflow to the oceanic regions is also strongest during the winter and that an aerosol build up here influences the atmospheric composition as well as climate over a larger region^44,45^. Therefore, the strategies, also taking into account the complex atmospheric dynamics besides the changes in emissions, are essential to reduce the aerosol build up and subsequently to mitigate their potential impacts on the air quality and climate, including the hydrological cycle. It should be noted that the focus of this study has been on the widespread and persistent enhancement which is clearly observed in the monthly mean distributions, although local sporadic emissions such as fireworks in the festive events have also been shown to increase the severity over Delhi^22,46^. Reductions in biomass-burning emissions would significantly improve the air quality of the IGP region during the post-monsoon^19,21,22,37,47,48^ but will have smaller effect in winter. A much more aggressive reduction of anthropogenic emissions over a wider area of IGP is required in the stagnant atmospheric conditions of winter to mitigate the PM_2.5_ enhancement caused by the meteorology and dynamics of the region. Strong dependence of surface PM_2.5_ on the meteorology simulated here using a regional model is in agreement with an earlier study based on global modeling over this region^23^. Among various anthropogenic sectors, residential energy usage is suggested to have larger effects on the annual mean PM_2.5_ in the region^31^. Future policies considering these would better complement the recent initiatives of the Indian government towards a reduction of household usage of biomass cook fuels^49,50^. Moreover, this study extends the possibilities of further evaluating health impacts due to changes in the individual aerosol constituents, originating from various biomass-burning and anthropogenic sources, besides the impact of overall PM_2.5_^11,31^.

Our study describes the widespread PM_2.5_ pollution over northern India during post-monsoon to winter and highlights the changing contributions from biomass-burning vs anthropogenic sources during the transition period. We also highlight a west-east gradient in terms of dominance of sources, with western cities dominated by biomass-burning sources and eastern cities dominated by anthropogenic sources during post-monsoon; this gradient weakens with the arrival of winter when the entire region from west to east is dominated by anthropogenic sources. We argue that a generic emission reduction policy will not yield desired results, and advocate for a season-based source-focused mitigation policy involving a reduction of anthropogenic emissions ubiquitously taking unfavorable regional dynamics into account, to improve the air quality and minimize the climate impacts.

## Methods

Weather Research and Forecasting model coupled with Chemistry (WRF-Chem)^33,51^, an online regional model version 3.8.1 has been used to simulate the meteorology and spatio-temporal distribution of PM_2.5_ over the northern Indian region. Model domain is centered at 76°E, 29°N and has 240 grid points in the east-west direction and 147 grid points in the north-south direction, along with 51 vertical levels. The simulations are conducted at a horizontal resolution of 12 km × 12 km. Anthropogenic emissions are included from the Southeast Asia Composition, Cloud, Climate Coupling Regional Study (SEAC4RS) for gas-phase species, except the NH_3_, which together with aerosols are included from the Hemispheric Transport of Air Pollution (HTAP) inventory^52^. The spatial resolution of these emissions is 0.1° × 0.1^o^. Biomass-burning emissions are based on the NCAR Fire Inventory (FINN)^53^ and biogenic emissions are calculated online using the Model of Emissions of Gases and Aerosols from Nature (MEGAN)^54^. Initial and lateral boundary conditions for the meteorological and chemical fields are included from the Era Interim and MOZART-4^55^ respectively. To limit the errors in simulated meteorology and dynamics, model is nudged to the Era Interim at all the vertical levels. Regional Acid Deposition Model - 2^nd^ generation (RADM2)^56^ coupled with the Modal Aerosol Dynamics Model for Europe/Secondary Organic Aerosol Model (MADE/SORGAM) aerosol module^57,58^ has been used to simulate the gas-phase chemistry and aerosols in the model. Application of this chemical mechanism showed small bias (~7%) in PM_2.5_ simulation over Europe^59,60^. The schemes opted to parameterize different processes in the model (Table S1) are based on earlier studies over this region using the WRF-Chem model^32,34,35^. A reduction in dust transport to 25% from the domain boundaries has been made resulting in relatively better agreement with the observations of AOD at 0.5 µm, nevertheless, no large changes were seen in the PM_2.5_ at the surface. Besides the reference simulation, two sensitivity simulations have been conducted (Table 1) by switching off the anthropogenic and biomass-burning emissions, one by one.

**Table 1:** Numerical simulations performed using the WRF-Chem model in the study.

Simulation name	Description
WRF-Chem or ref	Reference simulation driven by realistic emissions from anthropogenic, biogenic, and biomass-burning sources
fire_off	Biomass-burning emissions of all species turned off
anthro_off	Anthropogenic emissions of all species turned off

The observations of Aerosol optical depth (AOD) have been obtained from the MODIS (Moderate resolution Imaging Spectroradiometer) instrument onboard Aqua satellite, and MISR (Multi-angle Imaging Spectroradiometer) instrument onboard Terra satellite. Ground-based observations of AOD from the AERONET (Aerosol Robotic Network; https://aeronet.gsfc.nasa.gov/)^61^, at two stations (Kanpur and Jaipur) have been used. Fire count observations are obtained from the MODIS instrument to assess the biomass burning events. Ground-based observations of PM_2.5_ (µg m^−3^) at different stations in the IGP region (coordinates of stations provided in the Table S2) are obtained from the Central Pollution Control Board (CPCB), India (https://app.cpcbccr.com/ccr/#/caaqm-dashboard-all/caaqm-landing/data). The hourly datasets needed a screening for removal of fill values and also for very high values at few hours as discussed elsewhere^23^, performed before computing the daily average values. The HYSPLIT (HYbrid Single Particle Lagrangian Integrated Trajectory) model^62^ with meteorological inputs from the Global Data Assimilation System has been used to simulate the backward airmass trajectories.

## Supplementary information

Supplementary material.
