# Supplementary material for: On the widespread enhancement in fine particulate matter across the Indo-Gangetic Plain towards winter
Source: Sci Rep. 2020 Apr 3;10:5862. doi: 10.1038/s41598-020-62710-8 (PMC7125076; doi:10.1038/s41598-020-62710-8)
Supplement: Supplementary file 1 — Supplementary material. [file 41598_2020_62710_MOESM1_ESM.docx]

**Supplementary material**

**On the widespread enhancement in the fine particulate matter across the Indo-Gangetic Plain towards winter**

**Narendra Ojha^1^*, Amit Sharma^2, a^, Manish Kumar^3^, Imran Girach^4^, Tabish U. Ansari^5^, Som K. Sharma^1^, Narendra Singh^6^, Andrea Pozzer^3^, Sachin S. Gunthe^2^***

^1^Space and Atmospheric Sciences division, Physical Research Laboratory, Ahmedabad, India

^2^Department of Civil Engineering, IIT Madras, Chennai, India

^3^Atmospheric Chemistry Department, Max Planck Institute for Chemistry, Mainz, Germany

^4^Space Physics Laboratory, Vikram Sarabhai Space Centre, Thiruvananthapuram, India

^5^Lancaster Environment Centre, Lancaster University, UK

^6^Aryabhatta Research Institute of observational sciencES (ARIES), Nainital, India

^a^Now at: Laboratory for Atmospheric Research, Washington State University, Pullman, USA

***Correspondence:** N. Ojha ([ojha@prl.res.in](mailto:ojha@prl.res.in)) and S. S. Gunthe ([s.gunthe@iitm.ac.in](mailto:s.gunthe@iitm.ac.in))

**Table S1:** A list of different schemes opted to parameterize processes in the WRF-Chem model references

| **Process** | **Scheme used** |
| --- | --- |
| Cloud microphysics | Lin et al. scheme |
| Cumulus parameterization | Grell 3D Ensemble scheme |
| Land surface option | Unified Noah land surface model |
| Long wave radiation | Rapid Radiative Transfer Model |
| Short wave radiation | Goddard shortwave scheme |
| Planetary boundary layer | Mellor-Yamada-Janjic scheme |

**Table S2:** Observation sites of AOD (AERONET) and PM_2.5_ mass concentrations (µg m^-3^) (CPCB) in the northern Indian subcontinent used in this study for comparison with WRF-Chem results.

| **City** | **Observed parameter** | **Longitude** | **Latitude** |
| --- | --- | --- | --- |
| Kanpur | AOD | 80.23°E | 26.52°N |
| Jaipur | AOD | 75.81°E | 26.9°N |
| Delhi | PM_2.5_ | 77.27°E | 28.65°N |
| Lucknow | PM_2.5_ | 80.95°E | 26.85°N |
| Kanpur | PM_2.5_ | 80.33°E | 26.45°N |
| Varanasi | PM_2.5_ | 82.97°E | 25.32°N |
| Patna | PM_2.5_ | 85.08°E | 25.36°N |


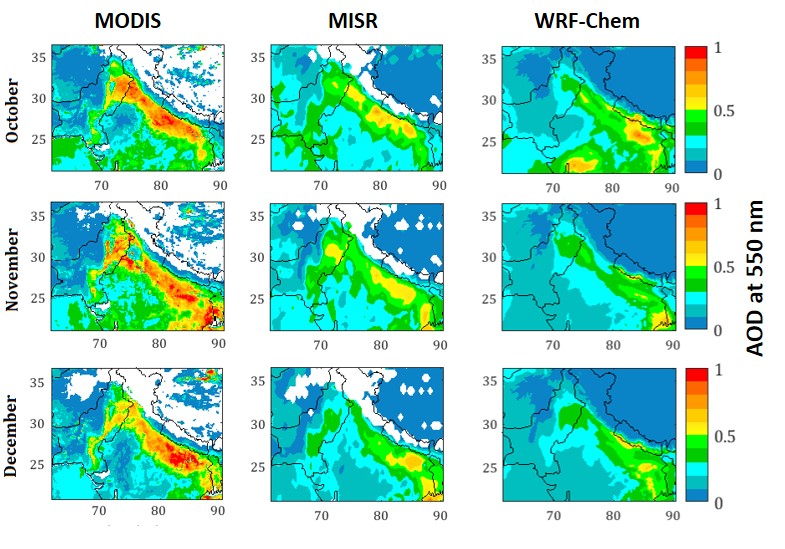


**Figure S1:** Spatial distribution of aerosol optical depth (AOD) at 550 nm observed from MODIS (1^st^ column), MISR (2^nd^ column), and simulated by WRF-Chem (3^rd^ column) during October, November, and December 2016.

**
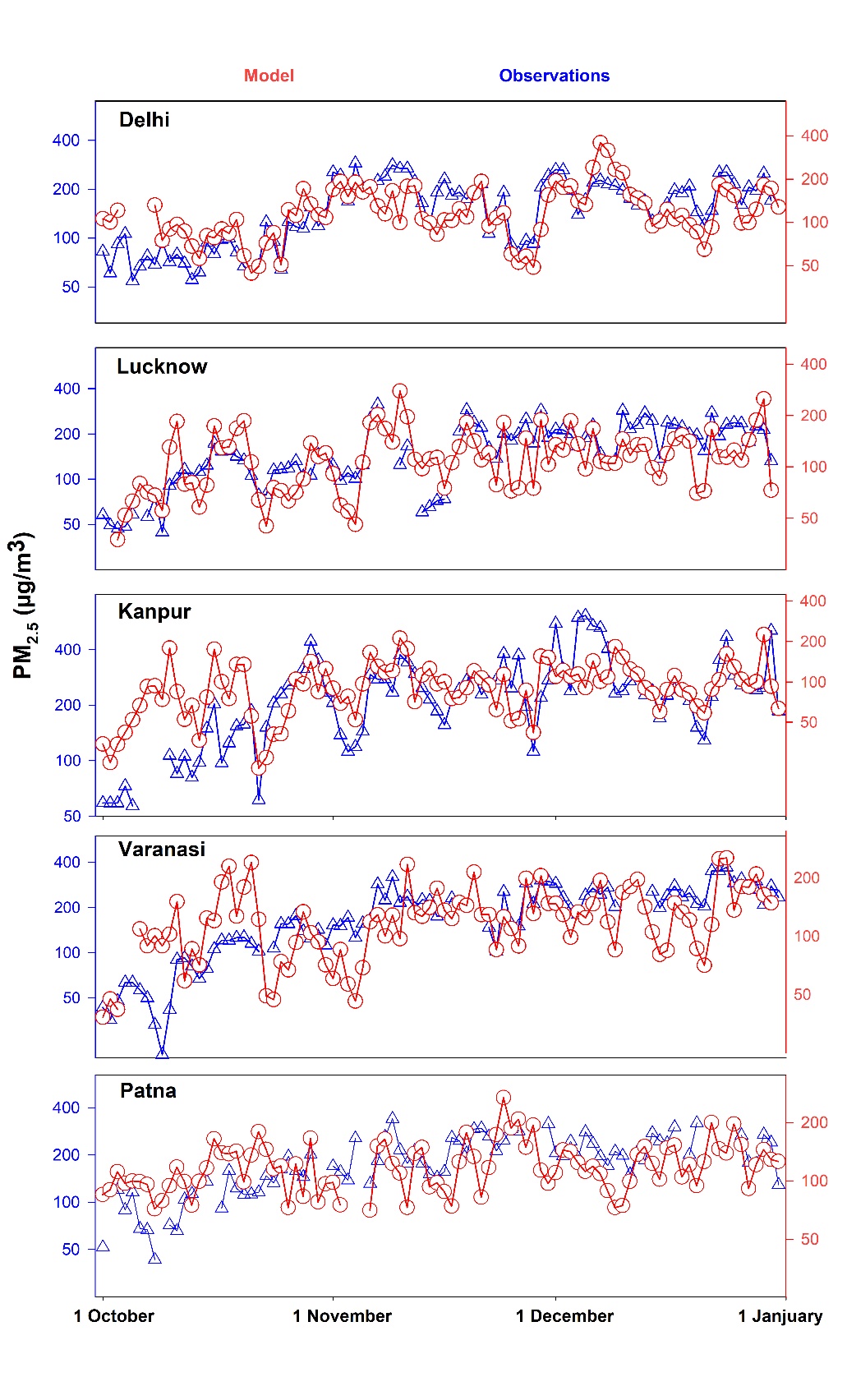
**

**Figure S2:** Time series of daily PM_2.5_ concentrations from the observations and model during October-December 2016. Note the difference in the range of observations and model, considering an underestimation by model (as discussed in the Section­– Aerosol Distribution: Model versus observations in the main manuscript).

**
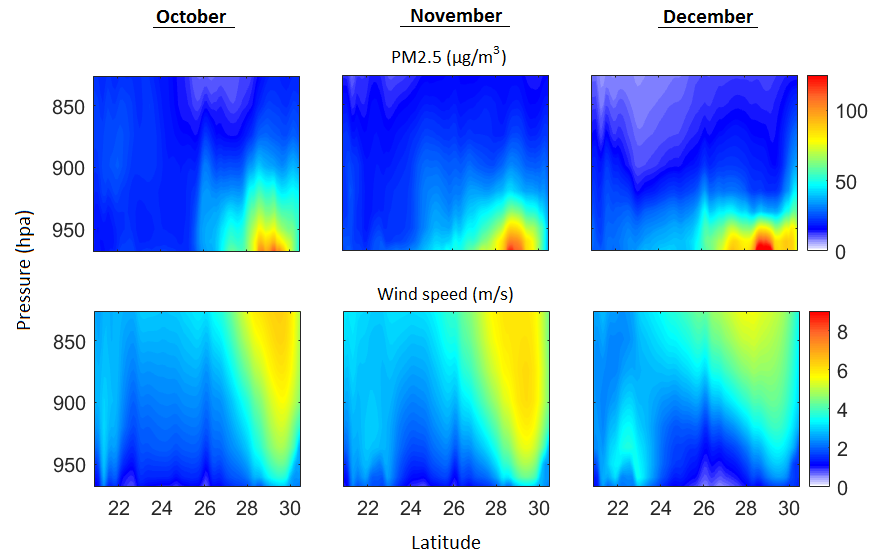
**

**Figure S3:** Vertical distribution of PM_2.5_ and wind speed with latitude (at 77 °E) during October-December 2016 simulated by the WRF-Chem model.

**
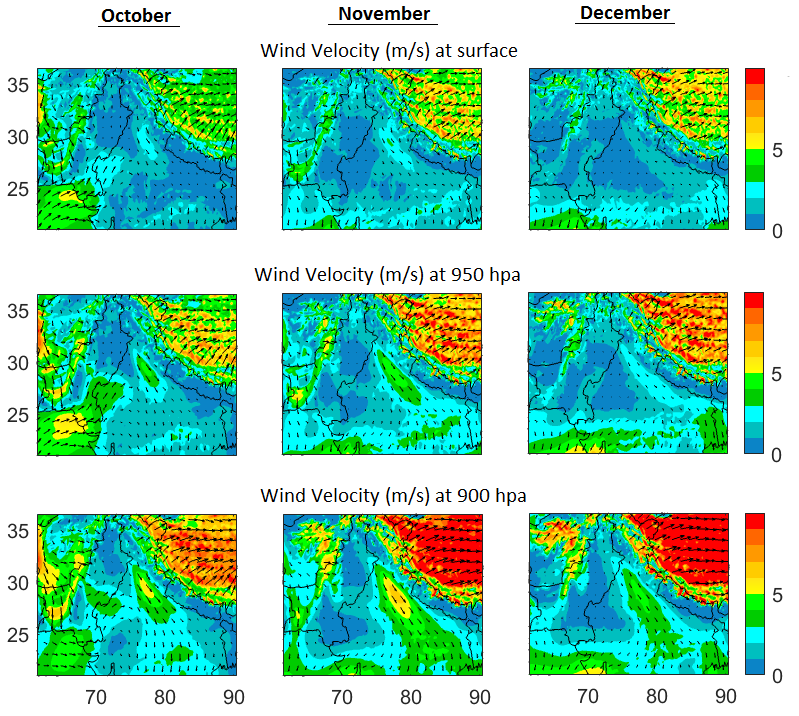
**

Figure S4: Mean winds (ms^-1^) at surface, ~950 hPa, and ~900 hPa over the northern Indian subcontinent during October, November and December 2016, based on the WRF-Chem model. Pressure is as a diagnostic (mean pressure profile over the region) and may not be representing actual pressures over high-altitude Himalaya and Tibetan Plateau.

**
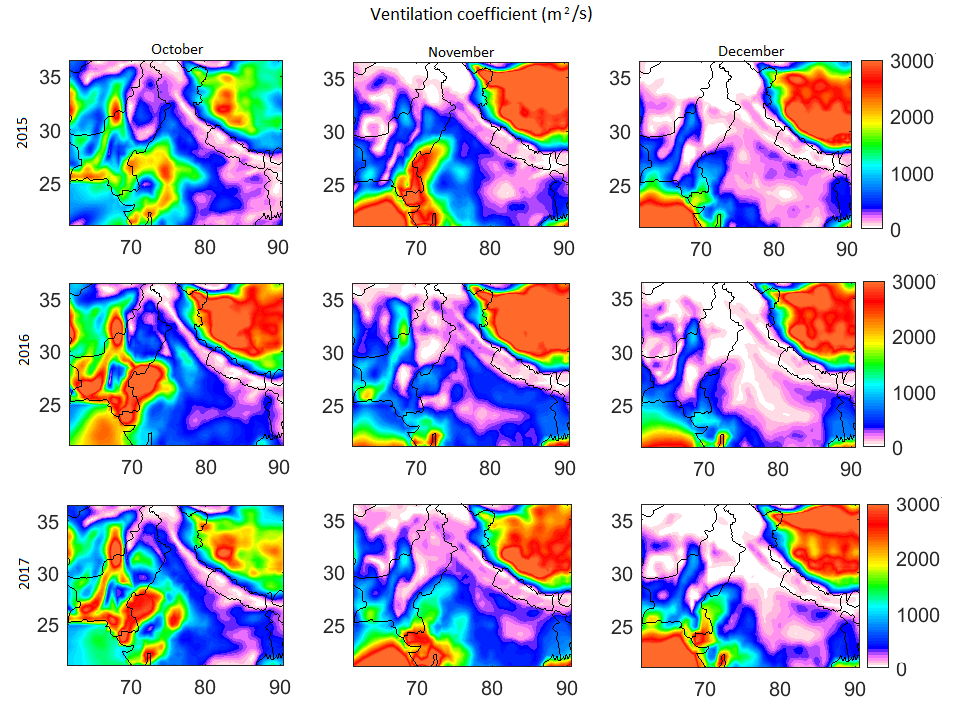
**

**Figure S5:** Ventilation coefficient (m^2^s^-1^) over the northern Indian subcontinent during October, November and December in years: 2015, 2016, and 2017 based on the Era Interim reanalysis.


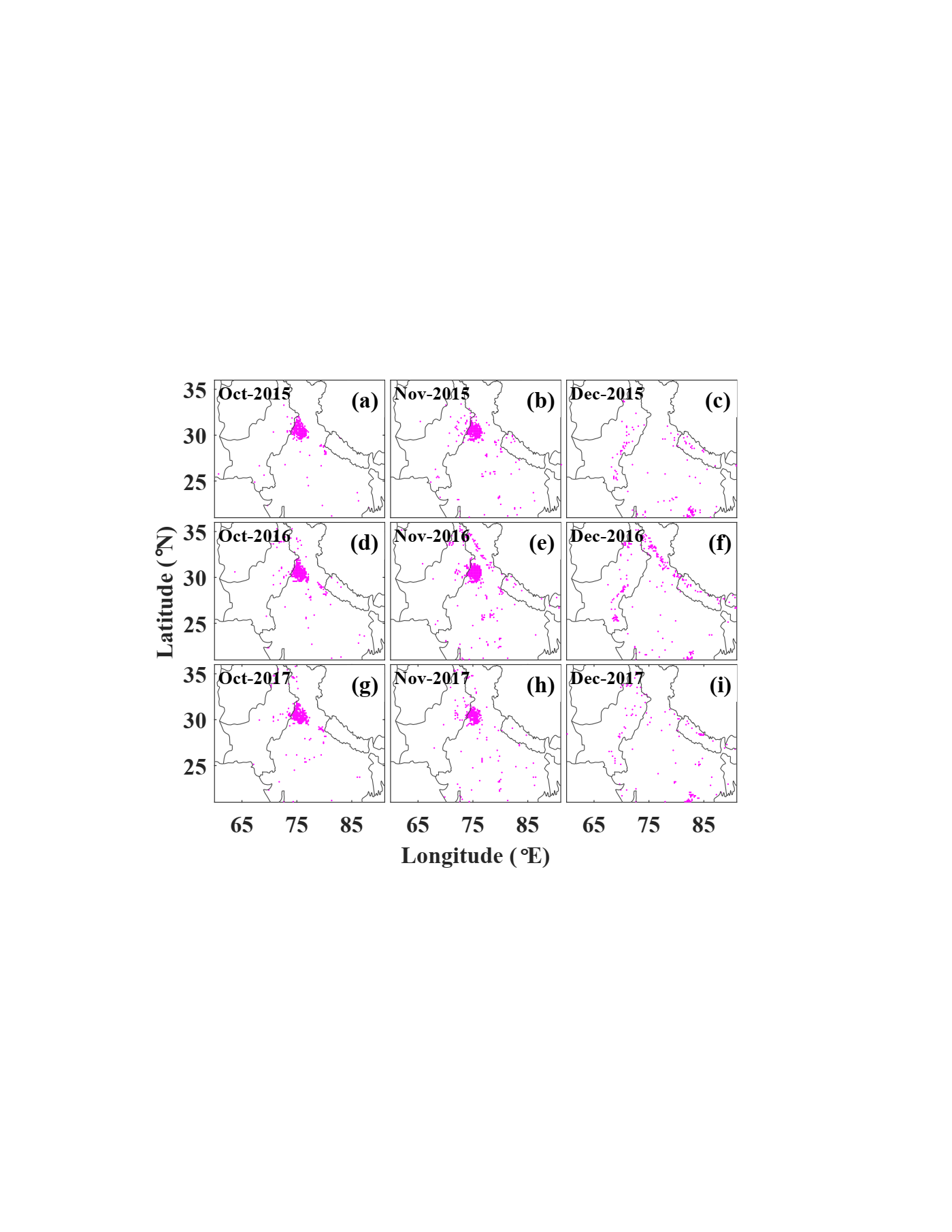


**Figure S6:** Fire locations (having fire detection confidence > 80%) over the northern Indian subcontinent during October, November, and December of years 2015, 2016, and 2017 based on the MODIS observations.

**
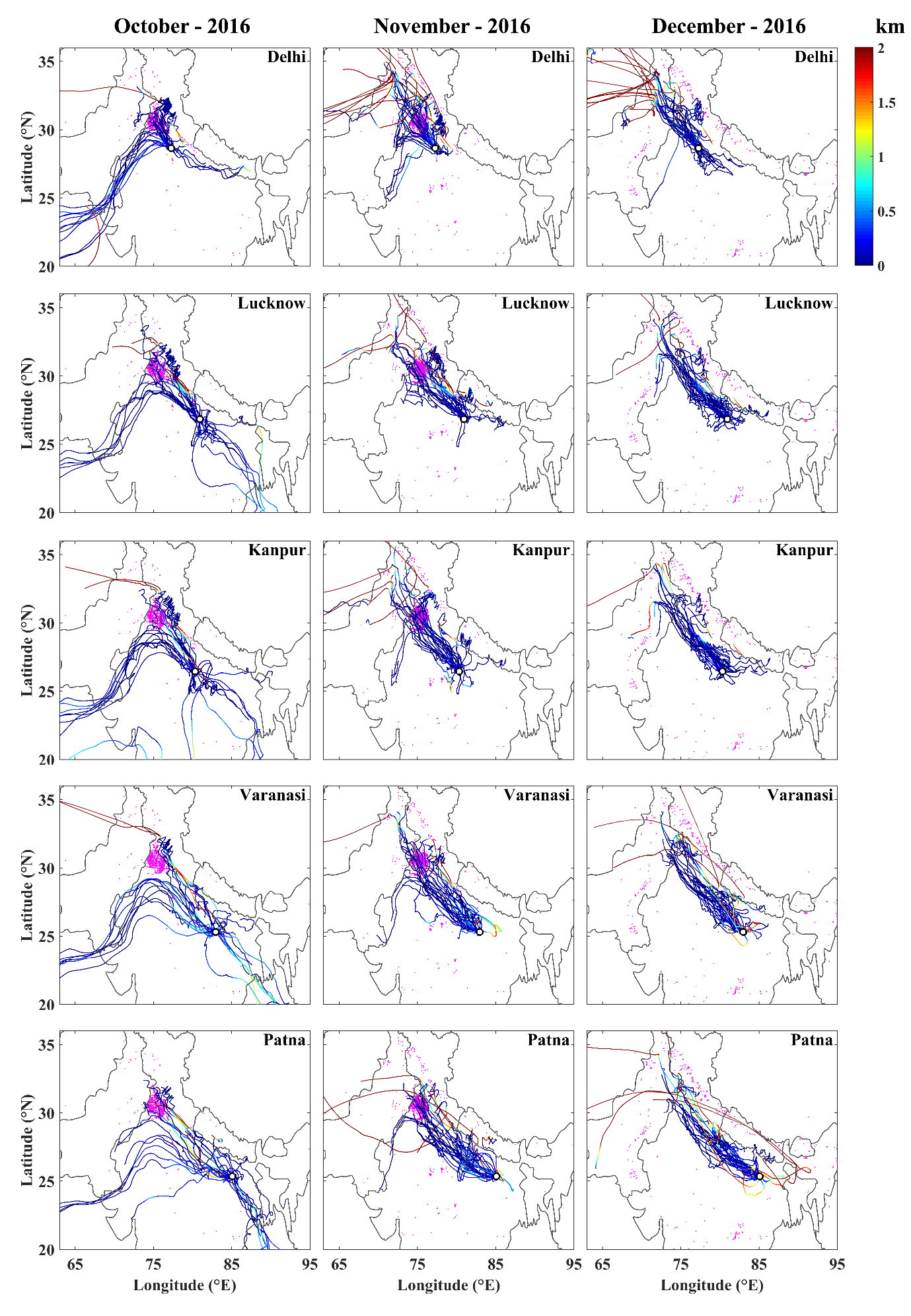
**

**Figure S7:** 7-day back airm trajectories at different stations above 200 m (agl) in the IGP region together with the MODIS fire locations during October, November, and December 2016. Colour along the trajectory shows altitude (km) of airmass above the ground level.


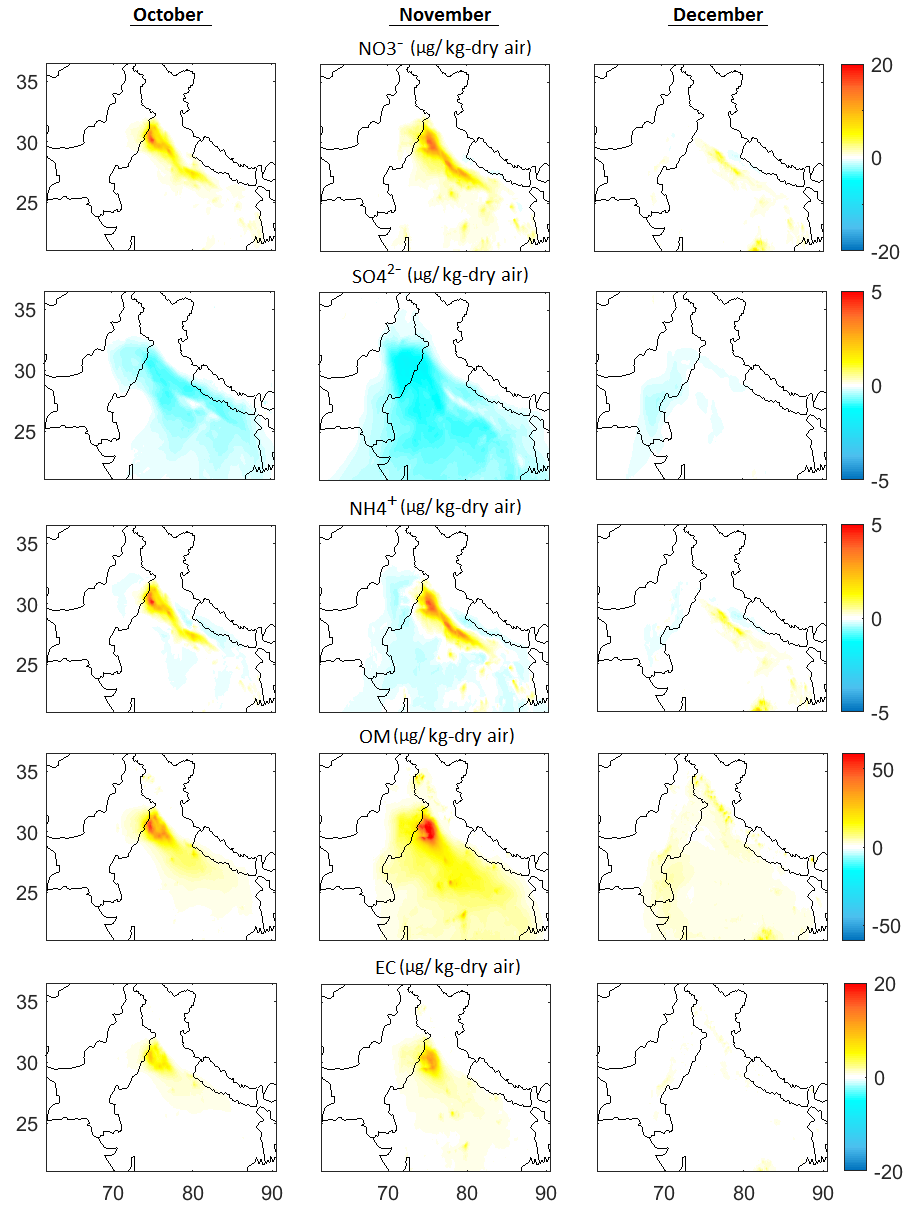


**Figure S8:** Spatial variation of difference (ref-fire_off) in the PM_2.5_ composition between WRF-Chem reference simulation and fire_off simulation during October, November and December 2016.


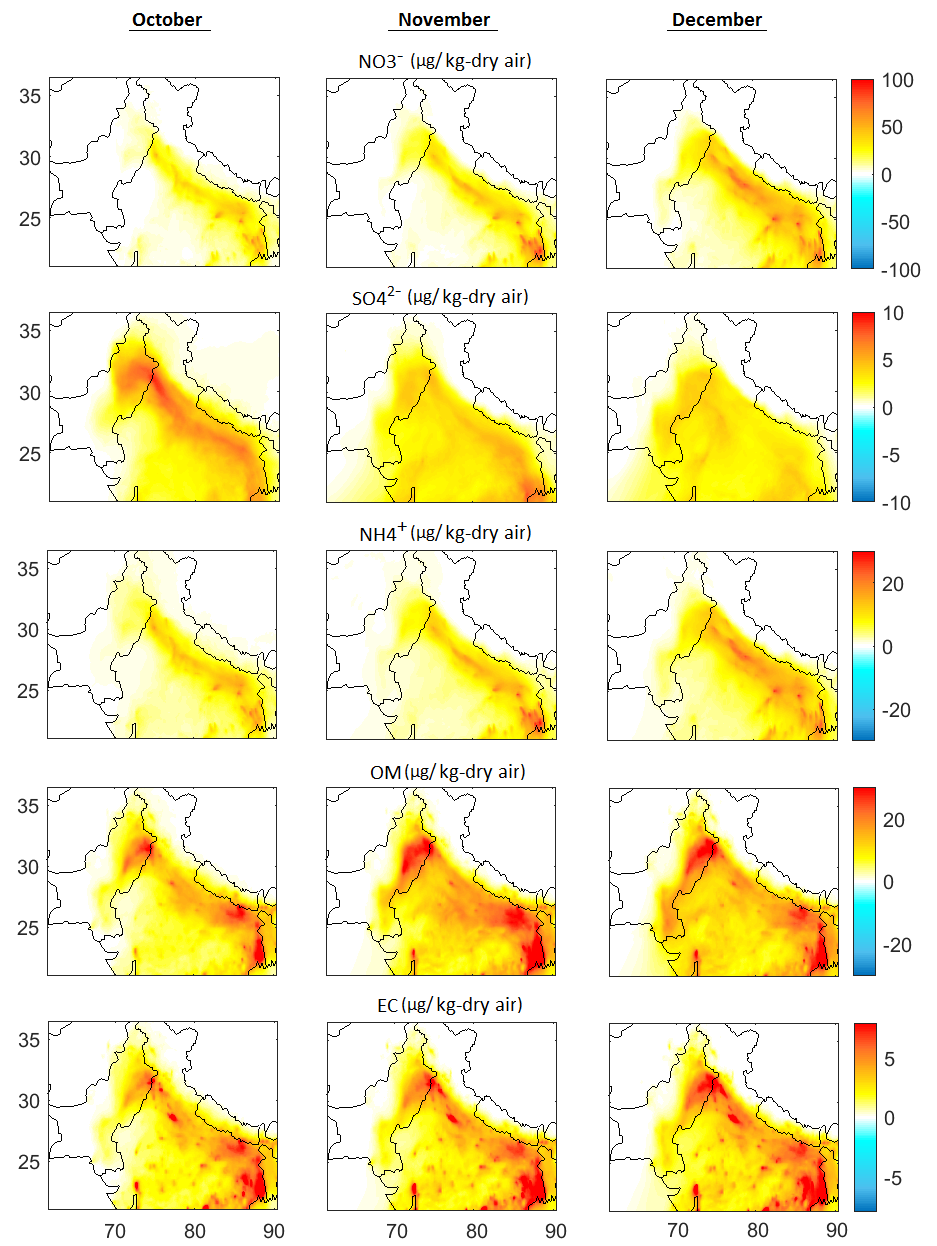


**Figure S9:** Spatial variation of difference (ref-anthro_off) in the PM_2.5_ composition between WRF-Chem reference simulation and anthro_off simulation during October, November and December 2016.
